# Disease profile and plasma neutralizing activity of post-vaccination Omicron BA.1 infection in Tianjin, China: a retrospective study

**DOI:** 10.1038/s41422-022-00674-2

**Published:** 2022-06-07

**Authors:** Hong Zheng, Yunlong Cao, Xiaosu Chen, Fengmei Wang, Ye Hu, Weiliang Song, Yangyang Chai, Qingqing Gu, Yansong Shi, Yingmei Feng, Shuxun Liu, Yan Xie, Xiaoliang Sunney Xie, Wentao Jiang, Zhongyang Shen

**Affiliations:** 1 Organ Transplant Center, NHC Key Laboratory for Critical Care Medicine, Tianjin First Central Hospital, Nankai University, Tianjin, China; 2 Changping Laborarty, Beijing, China; 3 Biomedical Pioneering Innovation Center (BIOPIC), Peking University, Beijing, China; 4 Frontier Research Center for Cell Response, Institute of Immunology, College of Life Sciences, Nankai University, Tianjin, China; 5 Institute of Hepatobiliary Disease, Tianjin Third Central Hospital, Tianjin, China; 6 Department of Immunology, Center for Immunotherapy, Institute of Basic Medical Sciences, Chinese Academy of Medical Sciences, Beijing, China; 7 Beijing Youan Hospital, Capital Medical University, Beijing, China; 8 National Key Laboratory of Medical Immunology, Institute of Immunology, Navy Medical University, Shanghai, China

**Keywords:** Immunology, Cell biology

Dear Editor,

SARS-CoV-2 Omicron variant BA.1 first emerged on the Chinese mainland on January 8, 2022, in Tianjin and caused a large wave of infections.^1^ Four rounds of mass testing of its ~14 million residents were launched on January 9, 12, 15, and 20, respectively. As of February 7, 2022, a total of 430 individuals were tested positive for Omicron BA.1, with no new infections detected for the following 16 days.

In the Chinese mainland, the most delivered vaccines are inactivated vaccines. As of January 8, 2022, up to 93.2% of Tianjin’s residents had been vaccinated with at least one dose.^2^ We investigated the protection conferred by vaccination against Omicron BA.1 by examining the breakthrough infections among vaccinees as compared with unvaccinated cases. Our study population comprised all 430 cases during the outbreak of Omicron BA.1 in Tianjin.

The demographic and clinical characteristics of the patients are summarized in Fig. 1a. The median age of the 430 patients was 36 (interquartile range (IQR), 14–55), with 26.5% under 18. Most patients presented with mild (47.7%) to moderate (50.2%) illness, with only 2 (0.5%) severe cases and no critical cases. No death was recorded. The most common onset symptoms were cough (37.0%) and fever (30.2%); parageusia (1.4%), heterosmia (0.9%), diarrhea (0.9%), and rash (0.2%) were rare (Supplementary information, Table S1). 40.0% of the patients had at least one comorbidity, with hypertension (17.0%) and abnormal liver function (16.0%) being the most common (Supplementary information, Table S2).

**Fig. 1:**
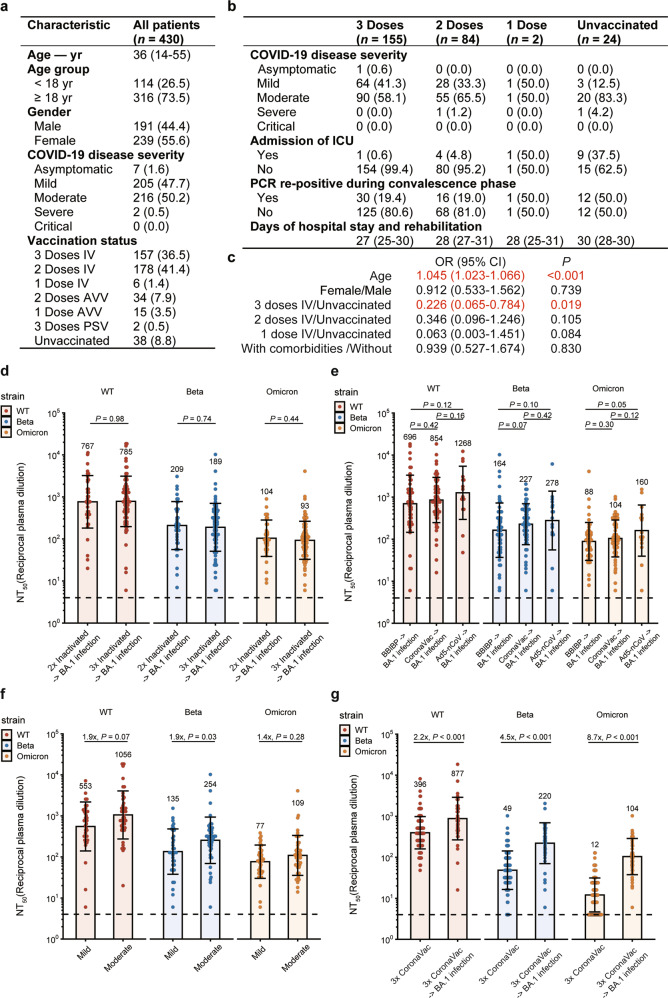
Protection of inactivated vaccine against Omicron BA.1. **a** Characteristics of Omicron-infected patients. Continuous variables were shown in median (IQR) and categorical variables were summarized as counts (percentages). COVID-19 disease severity was defined according to WHO living guidance for clinical management of COVID-19.^10^ IV, inactivated vaccine; AVV, adenovirus-vectored vaccine; PSV, recombinant protein subunit vaccine. **b** Correlation between inactivated vaccine doses and COVID-19 disease severity and progression in adult patients. PCR re-positive during convalescence phase was defined as PCR Ct value less than 40 after two independent PCR-negative results with an interval of more than 24 h. **c** Multivariate analysis of risk and protective factors for COVID-19 disease severity in Omicron-infected adult individuals. Statistical significance was determined by ordered multi-class logistic regression. **d** Humoral immune responses against WT and variants of SARS-CoV-2 among breakthrough Omicron convalescents. The geometric mean titer (GMT), geometric standard deviation, and fold-changes of 50% neutralization titers (NT_50_) are labeled. Dashed lines show the limit of detection. Statistical significance was determined by a two-tailed Wilcoxon test. **e** NT_50_ from the breakthrough BA.1 convalescents who were vaccinated with BBIBP-CorV (*n* = 54), CoronaVac (*n* = 65), or Ad5-nCoV (*n* = 16) vaccine. The plasma titers refer to all the recipients of the same type of vaccine, including 1-dose, 2-dose, and 3-dose vaccines. **f** NT_50_ from the breakthrough BA.1 convalescents with mild (*n* = 36) or moderate (*n* = 43) symptoms after 3 doses of inactive vaccine immunization. **g** NT_50_ from the breakthrough BA.1 convalescents who were vaccinated with 3 doses of CoronaVac vaccine (*n* = 42) or from the uninfected vaccinees who had matched vaccination profiles (*n* = 114).

Compared with adults, child patients presented with less severe illness. A vast majority (82.5%) of the child patients had mild symptoms with no severe cases. Adult patients were predominantly moderate cases (63.9%) (Supplementary information, Table S3). Notably, child patients presented with no ICU admission, fewer comorbidities (3.5% for children vs 53.2% for adults, *P* < 0.001), and less chance of turning re-positive on nucleic acid tests during convalescence phase (12.3% vs 22.5%, *P* = 0.019; Supplementary information, Table S3).

Of the 430 patients infected with Omicron BA.1, 341 (79.3%) received inactivated vaccines (54.3% BBIBP-CorV, 45.5% CoronaVac, and 0.3% other), 49 (11.4%) received adenovirus-vectored vaccines (Ad5-nCoV), 2 (0.5%) received recombinant protein subunit vaccine (ZF2001), and 38 (8.8%) were unvaccinated (Fig. 1a). Given that Tianjin launched the vaccination programs for children aged between 3 and 11 years old after October 30, 2021, most of the infected children (82.5%) only received 2 doses of inactivated vaccines (Supplementary information, Table S3).

Based on data from the 341 patients who had received inactivated vaccines (3-dose, *n* = 157; 2-dose, *n* = 178; 1-dose, *n* = 6) and 38 patients who had no SARS-CoV-2 vaccination, we found that when all age groups were considered together, 2 doses of inactivated vaccines were associated with a larger proportion of asymptomatic to mild disease compared with 3 doses (*P* = 0.003), which may be because most under-age patients had received 2 doses of inactivated vaccine and presented with mild illness (Supplementary information, Table S4). In adult patients alone, 3 doses reduced disease severity compared with no vaccination (*P* = 0.004, Fig. 1b).

ICU admission rate was only 0.6% for patients who had received a booster dose of inactivated vaccine and 4.8% for those who only received two-dose primary series, both significantly lower than the 37.5% for unvaccinated patients (*P* < 0.001, Fig. 1b).

Patients who had received a booster dose of inactivated vaccine experienced a shorter period of hospitalization and recovery. For patients across all age groups, the duration of hospitalization and recovery was 2 days shorter for those who had received 3 doses of inactivated vaccine than for unvaccinated patients (*P* = 0.009; Supplementary information, Table S4). For adult patients specifically, the duration of hospitalization and recovery for those who had received 3 doses (27 vs 30 days, *P* = 0.001) or 2 doses (28 vs 30 days, *P* = 0.026) of inactivated vaccine was shorter than unvaccinated patients (Fig. 1b). Compared with unvaccinated patients, receipt of 3 doses (19.4% vs 50.0%, *P* = 0.001) or 2 doses (19.0% vs 50.0%, *P* = 0.002) is also associated with lower re-positive rates during convalescence (Fig. 1b).

Vaccination status also correlated with immunity and inflammation-related laboratory findings (Supplementary information, Table S5). Compared with no vaccination, patients who received 3 doses of inactivated vaccines showed significantly lower levels of the systemic immune-inflammatory index (SII) and C-reactive protein during the early stage of recovery after nucleic acid tests turned negative, suggesting that receipt of inactivated vaccine can step up inflammation resolution. Due to relatively lower levels of lymphocytes, neutrophil/lymphocyte ratio (NLR), platelet/lymphocyte ratio (PLR), and monocyte/lymphocyte ratio (MLR) was higher in patients inoculated with three doses of inactivated vaccine than that of patients vaccinated with two doses. It has been reported that patients with severe COVID-19 are more prone to leukopenia and lymphopenia, and overly reduced lymphocyte percentage can be used as an indicator of disease severity.^3,4^ However, based on the overall immune characteristics of patients during the convalescence phase, we believe that these laboratory findings point to a lower degree of inflammation, suggesting that 3 doses of inactivated vaccine may shorten the course of illness by inducing resolution of inflammation. T-cell clustering indicated that the booster dose of inactivated vaccine led to a significant elevation of the CD4^+^/CD8^+^ ratio, Th1/Th2 ratio, and the ratio of activated Treg cells, suggesting that vaccination and infection may activate immune responses also by inducing spike-specific Th1 responses.^5, 6^ This implies that even if mutations on Omicron spike protein affect T-cell epitopes, immune responses mediated by T cells or non-neutralizing antibodies can still provide protection.^7, 8^ Tests of liver and kidney functions suggest that the number of inactivated vaccine doses did not affect liver and kidney function (Supplementary information, Table S6).

Ordered multi-class logistic regression model was constructed based on age, gender, number of vaccine doses, and comorbidities. In all age groups of patients, advanced age is a risk factor for severe disease (odds ratio (OR) 1.063, 95% CI 1.046–1.080, *P* < 0.001) (Supplementary information, Table S7). Among adult patients, age is an adverse factor for severe disease (OR 1.045, 95% CI 1.023–1.066, *P* < 0.001), while 3-dose inactivated vaccine is a protective factor (OR 0.226, 95% CI 0.065–0.784, *P* = 0.019, Fig. 1c). However, for child patients, no significant correlation was observed between age/gender/vaccination and disease severity (Supplementary information, Table S7).

Binary logistic regression suggests that receipt of 3 doses of inactivated vaccine is an independent protective factor against ICU admission for patients of all ages (OR 0.023, 95% CI 0.002–0.224, *P* = 0.001). The same applies to adult patients alone (OR 0.023, 95% CI 0.002–0.222, *P* = 0.001) (Supplementary information, Table S8). Receipt of 3 doses of inactivated vaccine is an independent protective factor against re-positive PCR during convalescence for patients of all ages (OR 0.317, 95% CI 0.144–0.700, *P* = 0.004) and adult patients (OR 0.301, 95% CI 0.117–0.771, *P* = 0.012) (Supplementary information, Table S9). Receipt of 3 inactivated vaccine doses is associated with shorter hospitalization and recovery (OR 0.461, 95% CI 0.225–0.946, *P* = 0.035), even when adjusted for age and gender. The same also applies to adult patients alone (OR 0.242, 95% CI 0.092–0.635, *P* = 0.004) (Supplementary information, Table S10).

To check whether BA.1 infection could induce a strong enough humoral immunity against Omicron, we obtained plasma samples from 135 Omicron convalescent patients 1 month after hospital discharge, including 60 mild cases and 75 moderate cases (Supplementary information, Table S11). We used authentic SARS-CoV-2 virus neutralization assays (CPE) to determine the plasma neutralizing antibody titers against WT, Beta, and Omicron BA.1. Overall, the geometric mean of 50% neutralizing titer (NT_50_) against WT was still higher than that against Omicron after BA.1 infection (Fig. 1d). Interestingly, the number of vaccine doses received before breakthrough infection does not significantly affect the NT_50_ after infection (Fig. 1d). Also, patients who had received the inactivated vaccine, BBIBP-CorV or CoronaVac, displayed a similar level of neutralizing antibody titers. Those who had received the adenovirus-vectored vaccine (Ad5-nCoV) showed slightly higher NT_50_ against WT, Beta, and Omicron BA.1; however, no statistical significance was achieved (Fig. 1e). Among patients who had received 3 doses of inactivated vaccines, the overall plasma neutralizing titer of moderate patients was higher than that of mild patients (Fig. 1f), consistent with the previous report.^9^

We compared the Omicron convalescent patients who had received 3 doses of CoronaVac (*n* = 42) with the healthy individuals who were also vaccinated with 3 doses of CoronaVac (*n* = 114) regarding the neutralizing antibody titers against WT, Beta, and Omicron BA.1. The healthy volunteers were selected to have a matched vaccination profile with the Omicron convalescents. For the 42 patients who had received 3 doses of CoronaVac, the median interval between receipt of the second dose and the third dose was 193.5 days (IQR 187.0–212.8); the median interval between receipt of the booster dose and infection was 55.5 days (IQR 37.8–75.0); the median interval between receipt of the booster dose and sampling was 95.5 days (IQR 80.5–116.8). For the healthy vaccinated cohort, the median interval between receipt of the second dose and the booster dose was 194.5 days (IQR 187.0–210.2); the median interval between receipt of the booster dose and sampling was 93.5 days (IQR 78.0–113.0). The geometric mean of the NT_50_ of Omicron convalescent patients was 2.2, 4.5, and 8.7 times that of the healthy vaccinated individuals when neutralizing WT, Beta, and Omicron BA.1, respectively (Fig. 1g). These observations suggest that infection with Omicron BA.1 could greatly elevate plasma neutralizing titers.

This study has several limitations. First, the sample of patients is small. Due to effective control efforts, the outbreak was soon contained, and the number of cases was thus limited. Second, the median interval between receipt of the final dose and infection is 79.5 (43.8–195.3) days among the adults and 48.0 (44.0–59.3) among children. This shorter interval may have attributed to the less severe symptoms observed in children, although a significant correlation between disease severity and vaccination-infection interval was not observed (Supplementary information, Table S12). Finally, since the vaccination-infection interval was different for the 2-dose and 3-dose groups, a comparison could not be conducted between the protective efficiency against severe diseases by three doses vs two doses.

## Supplementary information

Supplementary Information
