# Supplementary material for: Disease profile and plasma neutralizing activity of post-vaccination Omicron BA.1 infection in Tianjin, China: a retrospective study
Source: Cell Res. 2022 Jun 7;32(8):781–4. doi: 10.1038/s41422-022-00674-2 (PMC9171081; doi:10.1038/s41422-022-00674-2)
Supplement: Supplementary file 1 — Supplementary Information [file 41422_2022_674_MOESM1_ESM.pdf]

Disease profile and plasma neutralizing activity of post-vaccination Omicron BA.1 infection in Tianjin, China: a retrospective study

Hong Zheng[1,#], Yunlong Cao[2,3,#,*], Xiaosu Chen[4,#], Fengmei Wang[5], Ye Hu[4], Weiliang Song[3], Yangyang Chai[6], Qingqing Gu[2], Yansong Shi[4], Yingmei Feng[7], Shuxun Liu[8], Yan Xie[1], Xiaoliang Sunney Xie[2,3], Wentao Jiang[1], Zhongyang Shen[1,*]

[1]Organ Transplant Center, NHC Key Laboratory for Critical Care Medicine, Tianjin First Central Hospital, Nankai University, Tianjin, China

[2]Changping Laborarty, Beijing, China

[3]Biomedical Pioneering Innovation Center (BIOPIC), Peking University, Beijing, China

[4]Frontier Research Center for Cell Response, Institute of Immunology, College of Life Sciences, Nankai University, Tianjin, China

[5]Institute of Hepatobiliary Disease, Tianjin Third Central Hospital, Tianjin, China.

[6]Department of Immunology, Center for Immunotherapy, Institute of Basic Medical Sciences, Chinese Academy of Medical Sciences, Beijing, China

[7]Beijing Youan Hospital, Capital Medical University, Beijing, China

[8]National Key Laboratory of Medical Immunology, Institute of Immunology, Navy Medical University, Shanghai, China

[#]These authors contributed equally: Hong Zheng, Yunlong Cao, and Xiaosu Chen.

*Corresponding authors: Zhongyang Shen, Yunlong Cao

Supplementary information

Table of Contents:

Supplementary Information, Materials and Methods

References

Supplementary information, Tables

Table S1. Initial symptoms and convalescent symptoms of Omicron infected patients

Table S2. Comorbidities of Omicron infected patients

Table S3. Characteristics of Omicron infected patients

Table S4. Correlation between inactivated vaccine doses and COVID-19 disease severity and progression

Table S5. Laboratory Findings of Omicron infected patients

Table S6. Correlation between hepatic and renal function and vaccination

Table S7. Multivariate analysis of risk factors for COVID-19 disease severity

Table S8. Multivariate analysis of risk factors for ICU admission

Table S9. Multivariate analysis of risk factors for PCR re-positive during convalescence phase

Table S10. Multivariate analysis of risk factors for hospital stay and rehabilitation duration

Table S11. COVID-19 vaccination status of plasma samples prior to infection

Table S12. Correlation between COVID-19 disease severity and vaccination-infection interval

Supplementary Information, Materials and Methods

Data Sources

This study analyzed clinical and demographic data on the 430 Omicron patients reported by Tianjin Municipal Health Commission between January 8 and February 7, 2022, including COVID-19 vaccination history, laboratory tests, clinical symptoms, ICU admission and death. These data were extracted from the medical records obtained from Tianjin Haihe Hospital and Tianjin First Central Hospital where the patients received treatment after diagnosis.

Laboratory Confirmation

In this study, laboratory diagnosis was done in two designated COVID-19 hospitals: Tianjin Haihe Hospital and Tianjin First Central Hospital. Nucleic acid was extracted from respiratory samples using commercial kits (Zybio, 5203050). Reverse Transcription-Polymerase Chain Reaction (RT-PCR) was performed following the WHO protocol to detect two target genes, the open reading frame of 1ab (ORF1ab) and the nucleocapsid protein (N).5 ORF1ab forward primer (F): CCCTGTGGGTTTTACACTTAA, reverse primer (R): ACGATTGTGCATCAGCTGA, probe (P): 5'-FAM-CCGTCTGCGGTATGTGGAAAGGTTATGG-BHQ1-3’. N forward primer: GGGGAACTTCTCCTGCTAGAAT, reverse primer: CAGACATTTTGCTCTCAAGCTG, probe: 5'-FAM-TTGCTGCTGCTTGACAGATT-TAMRA-3'. 40 cycles of amplification (50°C for 10 min, 90°C for 5 min, 95°C for 10 s; 55°C for 40 s) were performed. A cycle threshold value (Ct-value) less than 37 with an S-shape amplification curve was defined as positive, and a Ct-value no less than 40 without a S-shape amplification curve was defined as negative. In the case of 37 ⩽ Ct < 40, retesting would be recommended. If both target genes (ORF1ab and N) are tested positive on real-time RT-PCR for the same sample, the result would be regarded as positive; if only one of the two target genes is tested positive, resampling and retesting would be required. If one of the two target genes is tested positive for two samples, the result would be positive.

Plasma Isolation

Peripheral blood was collected from 135 Omicron BA.1 convalescent patients 28 days after discharge and 114 healthy recipients of 3 doses of inactivated vaccines (CoronaVac) around 90 days after the booster shot. Whole blood samples were then diluted with PBS+2%FBS at 1:1 for Ficoll gradient centrifugation (Cytiva, 17-1440-03). Plasma was collected from the supernatants and restored at -80℃.

Authentic neutralization assay

A neutralization assay of authentic SARS-CoV-2 was performed using a cytopathic effect (CPE) assay. Briefly, various concentrations (2-fold serial dilution using DMEM) of plasma were mixed with the same volume of 100 PFU of authentic SARS-CoV-2 and incubated at 37°C for 1 h. The mixture was added to a monolayer of Vero-E6 cells (5x103 cells per well) in a 96-well plate and incubated for 1 h at 37°C. The supernatant was removed, and 200 μL of DMEM supplied with 2% (v/v) FBS was added to the infected cells. After incubation at 37°C supplied with 5% CO_2 for 5 days, all wells were examined for the CPE effect. All experiments were performed in a Biosafety Level 3 facility. Neutralization titers were calculated by the Spearman-Karber method.

Outcomes

The primary endpoints were COVID-19 severity and neutralizing antibody titers. COVID-19 disease severity was defined as asymptomatic, mild, moderate, severe and critical according to WHO living guidance for clinical management of COVID-19.

Secondary endpoints were intensive care unit (ICU) admission, re-positive results on nucleic acid tests during convalescence phase, and duration of hospitalization and recovery. PCR re-positive during convalescence phase was defined as PCR Ct value < 40 after two independent PCR-negative results at an interval of more than 24 hours. Duration of hospitalization and recovery was defined as the days spent in Tianjin Haihe Hospital and Tianjin First Central Hospital, respectively. All patients, including asymptomatic and mild cases, were hospitalized in Tianjin Haihe Hospital upon positive PCR results. Patients were discharged from Tianjin Haihe Hospital if the following criteria were met: 1) body temperature restored and stayed normal for over 3 days; 2) respiratory symptoms significantly relieved; 3) acute exudation substantially resolved on imaging study of the lungs; 4) negative on two consecutive PCR tests (at an interval of at least 24 hours) of samples collected from the respiratory tract. For patients whose PCR assays remained positive for over 4 weeks after criteria 1), 2) and 3) had been met, antibody assay and virus culture were applied to assess the risk of transmission before deciding whether these patients could be discharged.

Discharged patients from Tianjin Haihe Hospital were then transferred to Tianjin First Central Hospital for at least 14 days under medical observation. Patients received PCR assays on the 1st, 7th and 14th days after being transferred to Tianjin First Central Hospital. After 14 days of observation, patients with negative results on PCR and without other conditions in need of

hospitalization were discharged. Re-positive cases during convalescence phase were required to yield negative on consecutive PCR assays at an interval of at least 24 hours.

Study Oversight

This study was approved by the Tianjin Municipal Health Commission and the Ethics Committee of Tianjin First Central Hospital (Ethics committee archiving No. 2022N045KY). All patients/participants provided their written informed consent to have their clinical information collected for study purposes and the data generated from the study published. All the authors contributed to data collection and analysis, discussion and interpretation of the results. All the authors read and approved the final manuscript.

Statistical Analysis

Continuous variables were shown in medians and interquartile ranges (IQR), and the Mann-Whitney U test was used to analyze the differences between the two groups. Categorical variables were summarized as counts and percentages, and analyzed by Pearson's $\chi 2$ test. Ordered multi-class logistic regression model was used to analyze the relations between age/gender/receipt of inactivated vaccine and COVID-19 severity. No imputation was made for missing data. All the analyses were conducted using the SPSS software, version 22.0. Two-sided $P < 0.05$ was used in tests of significance.

Role of the funding source

The funders of the study had no role in study design, data collection, data analysis, data interpretation, or writing of the report.

Data sharing

Although all data used in this analysis were anonymized, the individual level nature of the data used risks individuals being identified, or being able to self-identify, if the data are released publicly. Extracted data are available upon request.

Ethical statement

This study was performed in accordance with Declaration of Helsinki after approved by the Tianjin Municipal Health Commission, and the Ethics Committee of Tianjin First Central Hospital (Ethics committee archiving No. 2022N045KY). Informed consent was obtained from all human research participants.

References

1 Tianjin Municipal Health Commission. http://wsjk.tj.gov.cn/ZTZL1/ZTZL750/YQFKZL9424/FKDT1207/202201/t20220109_5774785.html 2022.

2 Tianjin Municipal Health Commission. Vaccination of COVID-19 in Tianjin. http://wsjktjgovcn/ZTZL1/ZTZL750/YQFKZL9424/wjwymjzqk/202201/t20220109_5774994html 2022.

3 Fu L, Wang B, Yuan T *et al.* Clinical characteristics of coronavirus disease 2019 (COVID-19) in China: A systematic review and meta-analysis. *Journal of Infection* 2020; **80**:656-665.

4 Tan L, Wang Q, Zhang D *et al.* Lymphopenia predicts disease severity of COVID-19: a descriptive and predictive study. *Signal transduction and targeted therapy* 2020; **5**:33.

5 Swanson PA, 2nd, Padilla M, Hoyland W *et al.* AZD1222/ChAdOx1 nCoV-19 vaccination induces a polyfunctional spike protein-specific TH1 response with a diverse TCR repertoire. *Science translational medicine* 2021; **13**:eabj7211.

6 Painter MM, Mathew D, Goel RR *et al.* Rapid induction of antigen-specific CD4(+) T cells is associated with coordinated humoral and cellular immunity to SARS-CoV-2 mRNA vaccination. *Immunity* 2021; **54**:2133-2142.e2133.

7 Madhi SA, Ihekweazu C, Rees H, Pollard AJ. Decoupling of omicron variant infections and severe COVID-19. *Lancet (London, England)* 2022; **399**:1047-1048.

8 Milne G, Hames T, Scotton C *et al.* Does infection with or vaccination against SARS-CoV-2 lead to lasting immunity? *The Lancet Respiratory medicine* 2021; **9**:1450-1466.

9 Servellita V, Syed AM, Morris MK *et al.* Neutralizing immunity in vaccine breakthrough infections from the SARS-CoV-2 Omicron and Delta variants. *Cell* 2022; **185**:1539-1548.e1535.

10 WHO. Living guidance for clinical management of COVID-19: living guidance. https://apps.who.int/iris/handle/10665/349321 2021.

Table S1. Initial symptoms and convalescent symptoms of Omicron infected patients *

Symptoms	Initial	Convalescent
Cough	159 (37.0)	36 (8.4)
Fever	130 (30.2)	13 (3.0)
Sore throat	83 (19.3)	24 (5.6)
Fatigue	55 (12.8)	21 (4.9)
Nasal congestion	48 (11.2)	20 (4.7)
Nasal discharge	38 (8.8)	19 (4.4)
Parageusia	6 (1.4)	22 (5.1)
Heterosmia	4 (0.9)	22 (5.1)
Diarrhea	4 (0.9)	21 (4.9)
Rash	1 (0.2)	24 (5.6)
Conjunctivitis	0 (0.0)	22 (5.1)
Mucosal inflammation	0 (0.0)	21 (4.9)
Hypotension	0 (0.0)	22 (5.1)

* Categorical variables were summarized as counts (percentages).

Table S2. Comorbidities of Omicron infected patients *

**Disease**		
**Cardiovascular system**	Hypertension	73 (17.0)
	Coronary artery disease	18 (4.2)
	Arrhythmia	9 (2.1)
	Other	4 (0.9)
**Digestive system**	Abnormal liver function	69 (16.0)
	Fatty liver	17 (4.0)
	Gastrointestinal diseases	12 (2.8)
	Gallstone	7 (1.6)
	Other	7 (1.6)
**Endocrine system**	Diabetes	31 (7.2)
	Electrolyte disorder	20 (4.7)
	Hyperlipidemia	19 (4.4)
	Thyroid dysfunction	12 (2.8)
	Other	11 (2.6)
**Respiratory system**	Lung disease	20 (4.7)
	Bronchiectasia	3 (0.7)
**Nervous system**	Cerebrovascular disease	9 (2.1)
	Viral encephalitis	1 (0.2)
	Malignant tumor of brain	1 (0.2)
**Genitourinary system**	Chronic nephritis	3 (0.7)
	Renal malignancy	1 (0.2)
**Hematopathy**	Anemia	11 (2.6)
	Leucopenia	4 (0.9)
	Thrombocytopenia	3 (0.7)
	Leukemia	1 (0.2)
**Dermatosis**		3 (0.7)
**Other**		14 (3.3)

* Categorical variables were summarized as counts (percentages).

Table S3. Characteristics of Omicron infected patients *

Characteristic	All patients (*n* = 430)	Vaccination			Population		
		Vaccinated (*n* = 392)	Unvaccinated (*n* = 38)	*P*-value	Adult (*n* = 316)	Child † (*n* = 114)	*P*-value
**Age — yr**	36 (14-55)	36 (16-54.5)	30.5 (3-64)	0.616	47 (34-58)	10 (8-11)	< 0.001
**Gender**				0.325			0.162
Male	191 (44.4)	177 (45.2)	14 (36.8)		134 (42.4)	57 (50.0)	
Female	239 (55.6)	215 (54.8)	24 (63.2)		182 (57.6)	57 (50.0)	
**COVID-19 disease severity ‡**				0.188			< 0.001
Asymptomatic	7 (1.6)	6 (1.5)	1 (2.6)		1 (0.3)	6 (5.3)	
Mild	205 (47.7)	189 (48.2)	16 (42.1)		111 (35.1)	94 (82.5)	
Moderate	216 (50.2)	196 (50.0)	20 (52.6)		202 (63.9)	14 (12.3)	
Severe	2 (0.5)	1 (0.3)	1 (2.6)		2 (0.6)	0 (0.0)	
Critical	0 (0.0)	0 (0.0)	0 (0.0)		0 (0.0)	0 (0.0)	
**Vaccination status**				< 0.001			< 0.001
3 dose of IV	157 (36.5)	157 (40.1)	0 (0.0)		155 (49.1)	2 (1.8)	
2 dose of IV	178 (41.4)	178 (45.4)	0 (0.0)		84 (26.6)	94 (82.5)	
1 doses of IV	6 (1.4)	6 (1.5)	0 (0.0)		2 (0.6)	4 (3.5)	
2 dose of AVV	34 (7.9)	34 (8.7)	0 (0.0)		34 (10.8)	0 (0.0)	
1 dose of AVV	15 (3.5)	15 (3.8)	0 (0.0)		15 (4.7)	0 (0.0)	
3 doses of PSV	2 (0.5)	2 (0.5)	0 (0.0)		2 (0.6)	0 (0.0)	
Unvaccinated	38 (8.8)	(0.0)	38 (100.0)		24 (7.6)	14 (12.3)	
**Admission of ICU**				< 0.001			0.025
Yes	17 (4.0)	8 (2.0)	9 (23.7)		17 (5.4)	0 (0)	
No	413 (96.0)	384 (98.0)	29 (76.3)		299 (94.6)	114 (100.0)	
**Re-positive during convalescence phase §**				0.001			0.019
Yes	85 (19.8)	70 (17.9)	15 (39.5)		71 (22.5)	14 (12.3)	
No	345 (80.2)	322 (82.1)	23 (60.5)		245 (77.5)	100 (87.7)	
**Comorbidities**				0.001			< 0.001
None	258 (60.0)	239 (61.0)	19 (50.0)		148 (46.8)	110 (96.5)	
1	67 (15.6)	65 (16.6)	2 (5.3)		65 (20.6)	2 (1.7)	
2	40 (9.3)	37 (9.4)	3 (7.9)		39 (12.3)	1 (0.9)	
≥3	65 (15.1)	51 (13.0)	14 (36.8)		64 (20.3)	1 (0.9)	

* Continuous variables were shown in median (interquartile ranges), and the Mann-Whitney U test was used to analyze the differences between the two groups. Categorical variables were summarized as counts (percentages) and analyzed by Pearson's $\chi 2$ test. Percentages may not total 100 because of rounding.
† Child was defined as younger than 18 years old.
‡ COVID-19 disease severity was defined according to WHO living guidance for clinical management of COVID-19.
§ PCR re-positive during convalescence phase was defined as PCR Ct value less than 40 after two independent PCR-negative results with an interval of more than 24 hours.

Table S4. Correlation between inactivated vaccine doses and COVID-19 disease severity and progression *

	**All**				**Child †**			
	3 Doses (*n* = 157)	2 Doses (*n* = 178)	1 Dose (*n* = 6)	Unvaccinated (*n* = 38)	3 Doses (*n* = 2)	2 Doses (*n* = 94)	1 Dose (*n* = 4)	Unvaccinated (*n* = 14)
**COVID-19 disease severity ‡**								
Asymptomatic	1 (0.6)	5 (2.8)	0 (0.0)	1 (2.6)	0 (0.0)	5 (5.4)	0 (0.0)	1 (7.1)
Mild	66 (42.0)	104 (58.4)	4 (66.7)	16 (42.1)	2 (100.0)	76 (80.9)	3 (75.0)	13 (92.9)
Moderate	90 (57.3)	68 (38.2)	2 (33.3)	20 (52.6)	0 (0.0)	13 (13.8)	1 (25.0)	0 (0.0)
Severe	0 (0.0)	1 (0.6)	0 (0.0)	1 (2.6)	0 (0.0)	0 (0.0)	0 (0.0)	0 (0.0)
Critical	0 (0.0)	0 (0.0)	0 (0.0)	0 (0.0)	0 (0.0)	0 (0.0)	0 (0.0)	0 (0.0)
**Admission of ICU**								
Yes	1 (0.6)	4 (2.2)	1 (16.7)	9 (23.7)	0 (0.0)	0 (0.0)	0 (0.0)	0 (0.0)
No	156 (99.4)	174 (97.8)	5 (83.3)	29 (76.3)	2 (100.0)	94 (100.0)	4 (100.0)	14 (100.0)
**PCR re-positive during convalescence phase §**								
Yes	30 (19.1)	25 (14.0)	3 (50.0)	15 (39.5)	0 (0.0)	9 (9.6)	2 (50.0)	3 (21.4)
No	127 (80.9)	153 (86.0)	3 (50.0)	23 (60.5)	2 (100.0)	85 (90.4)	2 (50.0)	11 (78.6)
**Days of hospital stay and rehabilitation**								
	27 (25-30)	28 (26-30)	31 (30-31)	29 (26-33)	28 (26-30)	28 (25-30)	31 (31-31)	28 (26-29)

* Continuous variables were shown in median (interquartile ranges), and categorical variables were summarized as counts (percentages). Percentages may not total 100 because of rounding.

† Child was defined as younger than 18 years old.

‡ COVID-19 disease severity was defined according to WHO living guidance for clinical management of COVID-19.

§ PCR re-positive during convalescence phase was defined as PCR Ct value less than 40 after two independent PCR-negative results with an interval of more than 24 hours.

Table S5. Laboratory Findings of Omicron infected patients*

	3 Doses (*n* = 157)	2 Doses (*n* = 178)	1 Dose (*n* = 6)	Unvaccinated (*n* = 38)	*P*-value †	
**White-cell count — per mm^3**	6320 (5490-7230)	6310 (5300-7510)	7995 (7720-10680)	7420 (5130-8680)	0.016	3 V.S. 1: 0.004<br>2 V.S. 1: 0.009
**Lymphocyte count — per mm^3**	2080 (1650-2430)	2350 (1910-2900)	2930 (2640-3340)	2600 (1530-4090)	0.001	3 V.S.2: < 0.001<br>3 V.S.1: 0.031
**Neutrophil count — per mm^3**	3590 (2900-4230)	3260 (2440-4050)	4390 (3070-5720)	3590 (2510-4690)	0.045	3 V.S.2: 0.011
**Monocyte count — per mm^3**	410 (350-500)	410 (340-520)	530 (390-590)	490 (420-580)	0.021	3 V.S. Un 0.002<br>2 V.S. Un: 0.013
**Platelet count — per mm^3**	262,000 (227,000-307,000)	288,000 (238,000-326,000)	316,000 (306,000-329,000)	290,000 (237,000-386,000)	0.008	3 V.S.2: 0.010<br>3 V.S.1: 0.011
**SII**	372 (239-642)	492 (314-578)	390 (248-550)	468 (330-625)	0.032	3 V.S.2: 0.003
**NLR**	1.72 (1.41-2.11)	1.40 (1.00-1.96)	1.40 (1.03-1.72)	1.52 (0.93-2.36)	<0.001	3 V.S.2: < 0.001
**PLR**	131 (104-162)	114 (96-141)	102 (97-118)	107 (84-159)	0.031	3 V.S.2: 0.009
**MLR**	0.20 (0.17-0.24)	0.17 (0.14-0.22)	0.17 (0.12-0.23)	0.21 (0.14-0.30)	0.003	3 V.S.2: < 0.001
**C-reactive protein ≥10 mg/liter**					<0.001	3 V.S. Un: < 0.001<br>2 V.S. Un: 0.007
Yes	2 (1.3)	6 (3.4)	1 (16.7)	6 (16.2)		
No	155 (98.7)	171 (96.6)	5 (83.3)	31 (83.8)		
**Interleukin 6 ≥1.50 pg/milliliter**					0.309	
Yes	45 (28.7)	54 (30.5)	1 (16.7)	16 (43.2)		
No	112 (71.3)	123 (69.5)	5 (69.5)	21 (56.8)		
**CD4$^+$ T cell count — per microliter**	0.71(0.44-1.08)	0.75(0.47-1.15)	0.78(0.61-1.01)	0.65(0.39-1.16)	0.841	
**CD8$^+$ T cell count — per microliter**	0.77(0.48-1.30)	0.80(0.51-1.45)	0.48(0.34-3.32)	0.97(0.48-2.30)	0.508	
**CD4$^+$/CD8$^+$**	1.68(1.27-2.14)	1.47(1.17-1.78)	1.67(1.47-1.99)	1.56(1.35-2.29)	0.034	3 V.S.2: 0.034
**Treg cell count — per microliter**	3.41(2.65-4.32)	3.54(2.67-4.60)	4.10(2.79-6.98)	2.69(1.93-3.36)	0.116	
**Activated Treg cell count — per microliter**	1.22(0.76-1.67)	0.96(0.59-1.35)	1.82(0.75-2.71)	0.74(0.39-1.03)	0.003	3 V.S. 2: 0.044<br>3 V.S. Un: 0.035
**Th1 cell count — per microliter**	25.59(21.32-31.84)	22.17(17.96-27.86)	23.72(16.80-30.07)	28.23(21.05-33.22)	0.001	3 V.S. 2: 0.001
**Th2 cell count — per microliter**	49.23(39.66-56.55)	60.58(49.10-67.72)	50.01(20.44-68.58)	45.81(38.87-60.84)	<0.001	3 V.S. 2: < 0.001
**Th1/Th2**	0.55(0.38-0.79)	0.37(0.28-0.55)	0.59(0.24-1.60)	0.62(0.36-0.76)	<0.001	3 V.S. 2: < 0.001

* Data regarding white-cell, lymphocyte, neutrophil, monocyte, platelet count, SII, NLR, PLR, MLR, and the measurement of C-reactive protein and Interleukin 6 were missing for 2 patients (0.5%). Data were missing for the proportion of T cell subsets in 106 patients (28.0%). SII was defined as follows: SII = P x N/L, where P, N and L are the preoperative peripheral blood platelet, neutrophil and lymphocyte counts per liter, respectively. NLR was defined as the absolute neutrophil count divided by the absolute lymphocyte count. PLR was defined as the platelet count divided by the number of lymphocytes. MLR was defined as the ratio of absolute monocyte count to absolute

lymphocyte count. Continuous variables were shown in median (interquartile ranges). Statistical significance was determined by Kruskal-Wallis H test between groups and and Mann-Whitney U test between the two groups. Categorical variables were summarized as counts (percentages) and analyzed by Pearson's $\chi 2$ test. Percentages may not total 100 because of rounding.

† Only statistically significant results are shown here.

Table S6. Correlation between hepatic and renal function and vaccination*

	**3 Doses (*n* = 157)**	**2 Doses (*n* = 178)**	**1 Dose (*n* = 6)**	**Unvaccinated (*n* = 38)**	***P*-value**
Glutamic pyruvic transaminase — IU/liter	32.29 (20.32-67.33)	18.91 (12.53-40.79)	15.92 (12.06-28.17)	16.94 (13.85-29.29)	< 0.001
Glutamic oxalacetic transaminase — IU/liter	29.00 (23.14-37.88)	26.24 (22.96-34.05)	28.76 (27.16-31.73)	31.72 (25.79-39.11)	0.072
Total bilirubin — μmol/liter	12.33 (9.78-13.81)	11.68 (9.61-14.00)	-	10.62 (8.47-13.61)	0.322
Gamma-glutamyl transpeptidase — IU/liter	25.16 (17.33-54.26)	25.80 (15.14-51.63)	-	18.01 (14.47-38.44)	0.499
Alkaline phosphatase — IU/liter	68.12 (56.43-81.84)	74.89 (61.82-94.30)	-	79.67 (54.00-124.76)	0.133
Albumin — g/liter	42.73 (40.91-45.46)	41.43 (38.62-44.72)	-	36.12 (31.97-43.31)	0.008
Creatinine — μmol/liter	61.33 (50.86-71.81)	46.66 (38.31-58.80)	38.11 (37.61-59.12)	43.52 (32.30-58.82)	< 0.001
Urea nitrogen — mmol/liter	3.97 (3.35-4.69)	3.94 (3.10-4.68)	5.19 (4.06-5.65)	4.53 (3.48-5.87)	0.011
UREA/CREA †	0.07 (0.06-0.08)	0.08 (0.06-0.10)	0.12 (0.10-0.13)	0.11 (0.07-0.14)	< 0.001

* Data were missing for the measurement of glutamic pyruvic transaminase, glutamic oxalacetic transaminase, creatinine, urea nitrogen and UREA/CREA in 2 patients (0.5%), for total bilirubin, glutamyl transpeptidase, alkaline phosphatase and albumin in 322 patients (74.9%). Continuous variables were shown in median (interquartile ranges), and the Kruskal-Wallis H test was used to analyze the differences between groups.

† UREA/CREA was defined as the ratio of plasma urea nitrogen to plasma creatinine.

Table S7. Multivariate analysis of risk factors for COVID-19 disease severity*

	All		Child	
	**OR (95% CI)**	***P*-value**	**OR (95% CI)**	***P*-value**
**Age**	1.063 (1.046-1.080)	< 0.001	0.908 (0.755-1.093)	0.310
**Gender**				
Female	0.897 (0.566-1.422)	0.643	0.491 (0.175-1.374)	0.175
Male	Reference		Reference	
**Vaccination status**				
3 doses of IV	0.540 (0.220-1.326)	0.179	4.039 (0.052-314.191)	0.530
2 doses of IV	0.668 (0.278-1.600)	0.365	5.942 (0.799-44.124)	0.082
1 dose of IV	0.566 (0.061-5.217)	0.615	13.531 (0.829-220.964)	0.067
Unvaccinated	Reference		Reference	
**Comorbidities**				
With	1.014 (0.576-1.784)	0.962	3.216 (0.319-33.348)	0.319
Without	Reference		Reference	

* Statistical significance was determined by ordered multi-class logistic regression.

Table S8. Multivariate analysis of risk factors for ICU admission*

	All		Adult	
	**OR (95% CI)**	***P*-value**	**OR (95% CI)**	***P*-value**
**Age**	1.061 (1.012-1.112)	0.014	1.059 (1.009-1.112)	0.021
**Gender**				
Female	-		0.963 (0.223-4.155)	0.960
Male	Reference		Reference	
**Vaccination status**				
3 doses of IV	0.023 (0.002-0.224)	0.001	0.023 (0.002-0.222)	0.001
2 doses of IV	0.167 (0.038-0.739)	0.018	0.165 (0.037-0.732)	0.018
1 dose of IV	0.996 (0.045-21.970)	0.998	0.962 (0.038-24.604)	0.981
Unvaccinated	Reference		Reference	
**Comorbidities**				
With	30101052.05 (0.000-)	0.994	46178823.18 (0.000-)	0.996
Without	Reference		Reference	

* Statistical significance was determined by ordered multi-class logistic regression.

Table S9. Multivariate analysis of risk factors for PCR re-positive during convalescence phase*

	All		Adult	
	**OR (95% CI)**	***P*-value**	**OR (95% CI)**	***P*-value**
**Age**	1.019 (1.007-1.032)	0.002	1.023 (1.002-1.046)	0.035
**Gender**				
Female	-		1.457 (0.777-2.731)	0.241
Male	Reference		Reference	
**Vaccination status**				
3 doses of IV	0.317 (0.144-0.700)	0.004	0.301 (0.117-0.771)	0.012
2 doses of IV	0.297 (0.132-0.665)	0.003	0.279 (0.102-0.761)	0.013
1 dose of IV	1.985 (0.327-12.056)	0.456	1.201 (0.063-23.051)	0.903
Unvaccinated	Reference		Reference	
**Comorbidities**				
With	-		0.656 (0.329-1.308)	0.231
Without	Reference		Reference	

* Statistical significance was determined by ordered multi-class logistic regression.

Table S10. Multivariate analysis of risk factors for hospital stay and rehabilitation duration*

	All		Adult	
	**OR (95% CI)**	***P*-value**	**OR (95% CI)**	***P*-value**
**Age**	-		1.002 (0.985-1.020)	0.818
**Gender**				
Female	-		1.070 (0.642-1.780)	0.796
Male	Reference		Reference	
**Vaccination status**				
3 doses of IV	0.461 (0.225-0.946)	0.035	0.242 (0.092-0.635)	0.004
2 doses of IV	0.676 (0.334-1.367)	0.276	0.405 (0.149-1.097)	0.075
1 dose of IV	4.048 (0.431-38.029)	0.221	0.426 (0.023-8.032)	0.569
Unvaccinated	Reference		Reference	
**Comorbidities**				
With	-		0.992 (0.570-1.726)	0.992
Without	Reference		Reference	

* Statistical significance was determined by ordered multi-class logistic regression.

Table S11. COVID-19 vaccination status of plasma samples prior to infection *

	**3 doses of IV (*n* = 80)**	**2 doses of IV (*n* = 39)**	**2 doses of AVV (*n* = 16)**
CoronaVac	42 (52.5)	23 (59.0)	-
BBIBP-CorV	38 (47.5)	16 (41.0)	-
Ad5-nCoV	-	-	16 (100.0)

* Categorical variables were summarized as counts (percentages). IV, inactivated vaccine; AVV, adenovirus-vectored vaccine.

Table S12. Correlation between COVID-19 disease severity and vaccination-infection interval *

	**All kinds of vaccine**			**Inactivated vaccine**		
	All (*n* = 392)	Adult (*n* = 292)	Child (*n* = 100)	All (*n* = 341)	Adult (*n* = 241)	Child (*n* = 100)
**Severity**						
Asymptomatic	44.5 (43.3-46.5)	38	45 (44-47)	44.5 (43.3-46.5)	38	45 (44-47)
Mild	55 (45-136)	75.5 (43.5-182.5)	49 (45-64)	54 (45-136)	75 (45-182.5)	49 (45-64)
Moderate	78.5 (44-194.5)	81 (44.5-197)	47 (44-52)	75 (43-191)	80 (42-195)	47 (44-52)
Severe	172	172	-	172	172	-
Critical	-	-	-	-	-	-
***P*-value**	0.020	0.504	0.186	0.053	0.597	0.186

* COVID-19 disease severity was defined according to WHO living guidance for clinical management of COVID-19. Continuous variables were shown in median (interquartile ranges). Statistical significance was determined by Kruskal-Wallis H test between groups.
